# Long Non-coding RNA Structure and Function: Is There a Link?

**DOI:** 10.3389/fphys.2018.01201

**Published:** 2018-08-24

**Authors:** Anna Zampetaki, Andreas Albrecht, Kathleen Steinhofel

**Affiliations:** ^1^King’s British Heart Foundation Centre, King’s College London, London, United Kingdom; ^2^Faculty of Science and Technology, Middlesex University, London, United Kingdom; ^3^Department of Informatics, King’s College London, London, United Kingdom

**Keywords:** non-coding RNA, lncRNA, RNA structure, gene editing, cardiovascular diseases

## Abstract

RNA has emerged as the prime target for diagnostics, therapeutics and the development of personalized medicine. In particular, the non-coding RNAs (ncRNAs) that do not encode proteins, display remarkable biochemical versatility. They can fold into complex structures and interact with proteins, DNA and other RNAs, modulating the activity, DNA targets or partners of multiprotein complexes. Thus, ncRNAs confer regulatory plasticity and represent a new layer of epigenetic control that is dysregulated in disease. Intriguingly, for long non-coding RNAs (lncRNAs, >200 nucleotides length) structural conservation rather than nucleotide sequence conservation seems to be crucial for maintaining their function. LncRNAs tend to acquire complex secondary and tertiary structures and their functions only impose very subtle sequence constraints. In the present review we will discuss the biochemical assays that can be employed to determine the lncRNA structural configurations. The implications and challenges of linking function and lncRNA structure to design novel RNA therapeutic approaches will also be analyzed.

## Introduction

The HUMAN GENOME project has transformed our understanding of the basic unit of genetic information with RNA emerging as a versatile regulator of central cellular processes (Thum and Condorelli, 2015). The non-coding RNAs (ncRNAs), transcripts that do not encode proteins comprise the biggest class and are arbitrarily divided into small (<200 nucleotides) and long non-coding RNAs (lncRNA (>200 nucleotides). MicroRNAs (miRNAs) are the best studied small ncRNAs, representing an additional layer of posttranscriptional regulators that absorb perturbations and ensure the robustness of biological systems (Liu and Olson, 2010; Ebert and Sharp, 2012; Rotllan et al., 2016).

Substantial effort has now been directed toward dissecting the function of lncRNAs. In the cardiovascular system, lncRNAs were reported to play key roles in physiology and disease and targeting lncRNAs for novel therapeutic interventions has been explored (Uchida and Dimmeler, 2015; Boon et al., 2016; Buhrke et al., 2018). Here we will discuss the experimental tools to determine the RNA structure that can offer unique insights into the lncRNA function in the cardiovascular system.

## Challenges in Assessing lncRNA Functionality

The unique features of lncRNA have been extensive investigated (Guttman and Rinn, 2012; Ulitsky and Bartel, 2013; Bar et al., 2016; Ulitsky, 2016). Several characteristics of lncRNAs make functional evaluation challenging. Typically, lncRNAs display poor conservation across species showing only “patches” of conserved bases surrounded by large seemingly unconstrained sequences (Ponjavic et al., 2007; Guttman et al., 2009; Necsulea et al., 2014; Washietl et al., 2014). Additionally, lncRNAs exhibit low abundance that restricts their mode and sites of action (Mercer et al., 2008; Cabili et al., 2011, 2015; Washietl et al., 2014; Ulitsky, 2016; Wilk et al., 2016; Jandura and Krause, 2017). In terms of the modes of function, both *cis*-and *trans*-regulatory activity have been described (Mercer and Mattick, 2013). As *cis*-regulators, lncRNAs exert their function on neighboring genes on the same allele from which they are transcribed, displaying expression correlation and perturbation in an allele-specific manner. CARMEN, an enhancer associated lncRNA and a crucial regulator of cardiac specification in human cardiac progenitor cells was shown to act in *cis* to control the expression of miR-143/145 (Ounzain et al., 2015). On the other hand, acting in *trans-*lncRNAs can control gene expression at a distance from their transcription site, by altering the chromatin state, influencing the nuclear structure or regulating protein function (Vance and Ponting, 2014; Kopp and Mendell, 2018).

Intriguingly, for some low abundance lncRNAs the act of transcription seems to be more important than the transcript itself. In a seminal study, Engreitz et al. (2016) genetically manipulated 12 genomic loci that produce lncRNAs to find that 5 loci influenced the expression of a neighboring gene in *cis*. The expression of the lncRNAs transcripts themselves was not required but instead processes associated with their transcription were critical (Engreitz et al., 2016).

## The RNA Interactome

The above functional versatilities of lncRNAs stem from their ability to conform to different structures and molecular interactions with proteins, RNA and DNA (Guttman and Rinn, 2012; Marchese et al., 2017). In ribonucleoprotein complexes (RNPs), lncRNAs may act as scaffolds to stabilize the complexes, directing them to specific subcellular loci or the DNA. In endothelial cells, interaction of the lncRNA MANTIS with the ATPase catalytic subunits confers specificity to the switch/sucrose non-ferentable (SWI/SNF) chromatin remodeling complex directing it to a subset of angiogenic genes and facilitating nucleosome remodeling and transcription initiation (Leisegang et al., 2017; Zampetaki and Mayr, 2017). In fact binding of lncRNA to specific ATPase subunits of the SWI/SNF complex is a common regulatory mechanism (Cajigas et al., 2015; Zhu et al., 2016).

Interaction of lncRNAs with chromatin complexes is particularly important as these lncRNA-RNPs can trigger chromatin modifications through interference with the chromatin-modifying machinery (Tsai et al., 2010; Brockdorff, 2013; Simon et al., 2013). In the heart, Chaer a cardiac enriched lncRNA acts as an epigenetic switch by interfering with the polycomb repressive complex 2 (PRC2) and inhibiting H3K27m3 at genes involved in cardiac hypertrophy (Wang et al., 2016), while mesoderm faith determining lncRNA Fendrr can bind to both PRC2 and Trithorax group/MLL (TrxG/MLL) complexes acting as a fine tuner (Grote et al., 2013).

Apart from proteins, interaction of lncRNAs with DNA has also been described. This can lead to the formation of RNA-DNA triplex, a structure that is widespread *in vivo* and facilitates target gene recognition by lncRNAs (Mondal et al., 2015). This interaction was elegantly demonstrated in MEG3, a cardiac fibroblast enriched lncRNA that promotes fibrosis (Piccoli et al., 2017). MEG3 interacts with the PRC2 complex and forms RNA-DNA triplex structures through GA-rich sequence binding sites. Chromatin RNA immunoprecipitation revealed that MEG3 modulates the activity of TGF-b pathway genes and target recognition occurs via the triplex structures (Mondal et al., 2015).

Long non-coding RNA regulatory functions also rely on RNA–RNA interactions. Crosstalk with miRNAs creates an intricate network that exerts post-transcriptional regulation of gene expression. LncRNAs can harbor miRNA binding sites and act as molecular decoys or sponges that sequester miRNAs away from other transcripts. Noteworthy, competition between lncRNAs and miRNAs for binding to target mRNAs has been reported and leads to de-repression of gene expression (Yoon et al., 2014; Ballantyne et al., 2016). Finally, lncRNAs may contain embedded miRNA sequences and serve as a source of miRNAs (Piccoli et al., 2017).

## Linking RNA Structure to Function

RNA molecules adopt higher order tertiary interactions (Staple and Butcher, 2005; Wan et al., 2011). Although links between structure and function are emerging, the structural domains that dominate the RNA interactome are still not well defined. The functional implications of transcript structure are better understood in the processing the primary miRNAs (primiRNAs) to mature miRNAs. Using multiple mutagenesis assays, the secondary structures such as stem length, hairpin pairing, bulge size and position, and apical loop size that contribute to effective miRNA biogenesis were defined (Auyeung et al., 2013; Fang and Bartel, 2015; Nguyen et al., 2015; Roden et al., 2017). In clustered miRNAs that consist of multiple miRNA genes, the tertiary structure was also proposed to contribute to the processing to individual mature miRNAs. An autoregulatory role for the tertiary structure of miR-17∼92 cluster in its maturation and binding of auxiliary factors to conserved terminal loops was shown (Chakraborty et al., 2012). Recently, in the miR-497∼195 cluster, mutations in miR-195a hairpin were reported to affect the processing of miR-497a that resides in the same cluster. Computational analysis highlighted differences in the tertiary structure of the primiRNA in mutants that may affect the maturation process (Lataniotis et al., 2017). On a different note, in primiR-30c-1 the tertiary structure promotes the interaction with SRSF3, an SR protein family member that facilitates primiRNA recognition and processing. A single G/A sequence variation leads to a structural rearrangement of the apical region of the primiRNA affecting the conserved residues placed at the basal part of the stem and mature miRNA generation (Fernandez et al., 2017).

In lncRNAs, selection acting on structure rather than primary sequence may explain the rapid rate of evolution, that led to the “RNA modular code” hypothesis based on the view that selection acts on structural domains (Wutz et al., 2002; Tsai et al., 2010; Guttman and Rinn, 2012). Some experimental evidence supports this concept. The *MEG3* lncRNA gene contains three distinct structure modules M1, M2, and M3. Deletion analysis showed that motifs M2 and M3 are important for p53 activation. Intriguingly, a hybrid MEG3 transcript in which half of the primary sequence in the M2 motif was replaced by an entirely unrelated artificial sequence that displayed a similar secondary structure was fully functional in stimulating p53-mediated transcription (Zhang et al., 2010).

## RNA Structure Determination Methods

Chemical and enzymatic probing methods can provide an understanding of the secondary structure of RNA (Ehresmann et al., 1987). Enzymatic probing relies on nucleases that bind to paired and unpaired RNA and digest it to generate RNA fragments that can be analyzed. On the other hand, in chemical probing small size chemicals that react and covalently modify solvent accessible nucleotides are used. Following modification or cleavage, positions are typically mapped by reverse transcription, which either stops or introduces a mutation into the cDNA (Wilkinson et al., 2006). An analysis of the resulting cDNA is then used to determine the nucleotide position and modification frequency. Next generation sequencing (NGS) can be applied to directly sequence the cDNA products. This allows RNA structural characterization at a transcriptome-wide level in a single experiment (Lucks et al., 2011; Incarnato et al., 2014; Loughrey et al., 2014; Rouskin et al., 2014). Although initially the technologies were established to analyse RNA structure *in vitro*, structural characterization *in vivo* mainly through the use of probes that can diffuse quickly across membranes has also been reported (Spitale et al., 2013; Ding et al., 2014; Spitale et al., 2015; Flynn et al., 2016).

## Enzymatic Probing

### PARS

PARS (parallel analysis of RNA structure) is a high-throughput enzymatic probing method that measures the structural properties of isolated polyadenylated transcript pools that are renatured *in vitro* and treated with RNase V1 or S1. RNase V1 and RNase S1 cleave the 3′ phosphodiester bonds of double-stranded and single-stranded RNA, respectively, allowing evaluation of the double- or single-stranded conformation (Kertesz et al., 2010).

### Frag-Seq

Frag-Seq (fragmentation sequencing) is an enzymatic method that uses a nuclease P1 to specifically cleave single-stranded RNA. High-throughput sequencing then analyses the fragments generated. This workflow provides an “RNA accessibility profile” that is likened to the DNase hypersensitivity assays on chromatin (Underwood et al., 2010). Noteworthy, Frag-seq isolates fragments <200 bases after RNase P1 cleavage, hence large RNAs maybe underrepresented. As Frag-seq and PARS can provide complementary data a combined approach could improve the accuracy of genome-wide RNA structure measurements (Wan et al., 2011).

## Chemical Probing

### DMS Probing

The dimethyl sulfate (DMS) is a base specific reagent that can bind and alter the methylation state of unpaired adenosine and cytosine nucleotides (Tijerina et al., 2007; Rouskin et al., 2014). DMS footprinting is optimized for structural analysis of RNA. Protein binding to RNA generates a “footprint” that can be traced due to alterations in the RNA structure. The transcript size that can be evaluated is rather small (<500 nt) but this method can be performed both *in vitro* and *in vivo* as DMS can easily penetrate the cell membrane. DMS-seq that combines DMS methylation with NGS was recently performed *in vivo* (Ding et al., 2014; Rouskin et al., 2014).

### Targeted Structure-Seq

Targeted Structure-Seq relies on RNA methylation by DMS being performed *in vivo*. Subsequently, RNA is isolated from cells and the methylation sites are determined by employing gene specific primers for the reverse transcription reaction. Sequencing of the DMS derived fragments can be used to assess the cellular conformation of the RNA. Based on this method, structural models of elements within Xist were developed (Fang et al., 2015). Although initially reported using DMS this workflow can be adapted for other probing reagents.

### SHAPE

SHAPE (selective 2′-hydroxyl acylation by primer extension) can interrogate the RNA structure both *in vitro* and *in vivo* using the chemical NMIA and its derivatives to detect flexible regions in RNA secondary structure (Wilkinson et al., 2006; Weeks and Mauger, 2011). Several SHAPE reagents have been tested in order to improve the signal to background ratio (Lee et al., 2017). In SHAPE, the 2′-hydroxyl groups of all four nucleotides are selectively acylated when flexible and unpaired. This results in the formation of covalent SHAPE adducts that block the reverse transcription leading to truncated cDNA fragments. SHAPE reactivities can then be used to model secondary structures and quantify any process that modulates RNA dynamics.

### SHAPE-MaP

SHAPE-MaP (SHAPE and mutational profiling) was the first to combine the SHAPE protocol with NGS. Initially performed and reported to define the HIV-1 RNA genome, SHAPE-MaP is a highly sensitive technique that allowed rapid, *de novo* discovery and direct validation of new functional motifs (Siegfried et al., 2014; Mustoe et al., 2018).

### In-cell SHAPE-Seq

In-cell SHAPE-Seq is a modification of the SHAPE-Seq technique that combines the SHAPE-seq with gene expression measurements to elucidate the association of RNA structure and function *in vivo*. It revealed translational regulatory mechanisms in *E. coli* *in vivo* (Watters et al., 2016).

### icSHAPE-seq

icSHAPE-seq (*in vivo* click SHAPE sequencing) uses the in-cell SHAPE chemical NAI-N_3_ followed by selective chemical enrichment of NAI-N_3_-modified RNA that provides an improved signal-to-noise ratio (Flynn et al., 2016). Follow-up NGS allows accurate identification at single-nucleotide resolution. In mouse embryonic stem cells it was shown that *in vitro* RNA folding is programmed entirely by the sequence, whereas *in vivo*, the RNA structure depends on the context of intracellular environment and interaction with RNA binding proteins that may lead to focal structural rearrangements (Spitale et al., 2015). Hence, this assay offers the exciting possibility of viewing the RNA structurome *in vivo* in the presence or absence of stimulation.

## RNA Structurome and Interactome Determination

### PARIS

PARIS (psoralen analysis of RNA interactions and structures) was recently developed to determine both RNA structure and interactions *in vivo*. It uses the highly specific and reversible nucleic acid crosslinker psoralen-derivative 4′-aminomethyltrioxsalen to fix base pairs in living cells. Subsequently, partial RNase and complete proteinase digestion lead to purification of a set of small crosslinked and directly base-paired RNA fragments. Purification of the crosslinked fragments using 2D electrophoresis, followed by proximity ligation of duplex RNA fragments, reversal of crosslinks, and high throughput sequencing reveals the direct base pairing between fragments. Based on these reads, models of RNA structures and interactions can be generated with high specificity and sensitivity (Lu et al., 2016). Using this approach a model for the higher order structure of Xist was interrogated (Lu et al., 2016). Encouragingly, these findings are in agreement with crystallographic studies of the defined domains *in vitro* (Arieti et al., 2014).

## lncRNA Structure Determination

Structure determination of lncRNA *in vivo* is extremely challenging as they are highly heterogenic with regions with well-defined base-pairing, others without base-pairing and regions with multiple structures. Additionally, lncRNAs may stretch across thousands of nucleotides, they are expressed in low abundance and tend to be part of multicomponent complexes (Busan and Weeks, 2017). Nevertheless, the structure of several lncRNAs has been experimentally determined (**Table 1**).

**Table 1:** Structural Determination of lncRNAs.

LncRNA (size)	Mode of action	Function	Structure	Probing techniques	References
Xist (17,000 nucleotides)	*cis*	X-chromosome inactivation.	Regions A-F with distinct repeat sequences.	*In vivo* and *in vitro* SHAPE-MaP. Targeted structure Seq.PARIS	Simon et al., 2013; Fang et al., 2015; Lu et al., 2016; Smola et al., 2016
RepA (1,600 nucleotides)	*cis*	Encoded by an internal promoter on the Xist gene sense strand.	Three folding modules.	*In vitro* using chemical probing with SHAPE and DMS reagents.	Liu et al., 2017a
Rox1 (3,700 nucleotides) Rox2 (1,200 nucleotides)	*cis* and *trans*	Male specific nuclear RNAs. Dosage compensation.	Rox1: three stable helices connected by flexible linker regions. Rox2: two clusters of tandem stem-loops.	*In vitro* using chemical probing with SHAPE and PARS analysis. Both methods independently support the rox2 structure model.	Ilik et al., 2013
SRA (870 nucleotides)	*trans*	Interacts with SRA protein to regulate cardiac muscle differentiation.	Four distinct domains.	*In vitro* SHAPE and DMS chemical probing. Good agreement with RNase V1 enzymatic probing.	Novikova et al., 2012
HOTAIR (2,148 nucleotides)	*trans*	Associated with sporadic thoracic aortic aneurysm and non-end stage heart failure. Circulating biomarker for acute myocardial infarction and congenital heart diseases.	Four structural modules.	*In vitro* using chemical probing with SHAPE and DMS reagents.	Somarowthu et al., 2015; Greco et al., 2016; Gao et al., 2017; Guo et al., 2017; Jiang et al., 2018
Braveheart (590 nucleotides)	*trans*	Cardiovascular lineage commitment.	Three domains. Critical structure: a 5′ asymmetric G-rich internal loop (AGIL).	*In vitro* using chemical probing with SHAPE and DMS reagents.	Xue et al., 2016


### Xist

This is a very long lncRNA (17,000 nucleotides) controlling X chromosome inactivation. It spreads across the entire chromosome while triggering stable epigenetic modifications through recruitment of the PRC2 complex and enrichment for the H3K27me3 repressive chromatin modification (Simon et al., 2013; Fang et al., 2015; Smola et al., 2016). *In vivo* SHAPE data identified 33 regions in Xist that form well-defined secondary structures linked by structurally variable and dynamic regions.

### RepA

This is a 1,600 nucleotides mouse lncRNA encoded by an internal promoter on the Xist-gene sense strand. Applying SHAPE and DMS chemical probing *in vitro*, an intricate structure of three independently folding modules was revealed. Phylogenetic analysis and computational 3D modeling demonstrated a defined tertiary architecture that can form autonomously in the absence of protein partners (Liu et al., 2017a).

### Rox1/Rox2

In Drosophila dosage compensation is achieved using two lncRNAs that are transcribed from the X chromosome. RNA on the X 1 and 2 (roX1 and roX2) are 3,700 and 1,200 nucleotides in length, respectively. *In vitro* SHAPE probing and PARS analysis revealed common, conserved and distinct structural motifs that may function as targeting sites and assembly platforms for the male specific lethal complex (Ilik et al., 2013).

### SRA

The human steroid receptor RNA activator (SRA) is an 870 nucleotide lncRNA that is derived from a gene encoding both lncRNA and protein coding transcripts. The structure of SRA was experimentally interrogated using SHAPE and DMS chemical probing *in vitro*. In parallel, RNase V1 enzymatic probing was performed. It was shown that SRA consists of four distinct domains with a variety of secondary structures (Novikova et al., 2012). More importantly, comparative structural analysis between mouse and human strongly suggested that a large number of evolutionary changes had minimal mutational effect on the protein derived from the locus while stabilizing the RNA structural core (Novikova et al., 2012).

### HOTAIR

Associated with Sporadic Thoracic Aortic Aneurysm through regulation of extracellular matrix deposition and human aortic smooth muscle cells apoptosis, this lncRNA plays a key role in the cardiovascular system (Guo et al., 2017). In non-end stage heart failure patients HOTAIR was among a panel of lncRNAs that were significantly modulated (Greco et al., 2016). A protective role of HOTAIR in cardiomyocytes (Gao et al., 2017) and as a circulating biomarker for acute myocardial infarction and congenital heart diseases were also proposed (Jiang et al., 2018). Hotair is 2,148 nucleotides long making the structural determination extremely challenging. To address this issue a non-denaturing purification protocol to obtain a homogeneous and monodisperse form was established. Structural modules and distinct evolutionary conserved elements were determined *in vitro* using chemical probing with SHAPE and DMS reagents (Somarowthu et al., 2015).

### Braveheart

*Braveheart* is a 590 nucleotide lncRNA that acts in *trans* to regulate cardiovascular lineage commitment. Its secondary structure was experimentally assessed using SHAPE and DMS probing *in vitro*. It emerged that *Braveheart* is organized into a highly intricate modular structure comprising of three domains, consisting of 12 helices, 8 terminal loops, 5 sizeable internal loops, and a five-way junction. Intriguingly, it includes a 5′ asymmetric G-rich internal loop (AGIL) and a 55 nucleotide stretch at the 3′ end exhibiting high reactivity suggesting low probability of structure. Genetic deletion of this specific 11 nucleotide fragment demonstrated that the AGIL motif is essential for mouse embryonic stem cell differentiation to cardiomyocytes through binding of the zing-finger protein CNBP/ZNF9 (Xue et al., 2016).

## Future Directions

RNA structure determination combined with genetic manipulations can elucidate the important functional domains of lncRNAs. To this end, advanced experimental tools, bioinformatics and genome engineering should be integrated. The CRISPR/Cas9 gene editing system emerged as a robust technology that can be used to generate targeted modifications at precise genomic loci. Cas9, a nuclease that can induce double-stranded breaks (DSBs) to the DNA, can be guided in the immediate vicinity of the proto-adjacent motif NGG by an RNA molecule (sgRNA) consisting of a small 20 nucleotide long variable sequence and an adaptor transactivating RNA. Precise insertions, deletions, or base substitutions can be introduced at a DSB site (Lin et al., 2014) in primary cells and *in vivo* in mouse models of disease (Platt et al., 2014; Abrahimi et al., 2015). A modified version of the CRISPR/Cas9 system has recently been employed for genome scale screenings of functional lncRNAs. This CRISPR interference approach uses a nuclease dead Cas9 (dCas9) that is not capable of inducing DSB to the DNA. Fused to a repressor domain (e.g., KRAB) (Liu et al., 2017b) or an activation domain (e.g., VP64) (Konermann et al., 2015; Bester et al., 2018) dCas9 and can be guided by sgRNAs to specific loci in the upstream regulatory region to trigger repression or activation of lncRNA transcription, respectively. Such approaches are extremely useful to test the functionality of lncRNAs in a high throughput manner.

Once specific lncRNAs are identified, technologies that can define the lncRNA structure *in vivo* are critical to determine lncRNA modules and structural domains. RNA structure determination can be coupled with comparative genomics analysis that will take into consideration the positional conservation and the fact that lncRNAs may rely on short elements rather than long stretches of conserved sequences. Genetic studies that can target precisely these structural domains while maintaining the expression of the lncRNA (Matsumoto et al., 2017) will delineate the functional impact of these motifs. The use of CRISPR/Cas9 gene editing in induced pluripotent stem cells that can be clonally expanded, engineered to harbor defined deletions of the structural motifs and differentiated to other cell types (Cochrane et al., 2017; Granata et al., 2017) can provide conclusive evidence for the functional impact of these domains in the cardiovascular system. The potential of these elements as novel targets could be explored further for precise interventions suitable for therapeutic applications.

## Author Contributions

AZ initiated the study, designed its structure, and wrote the manuscript. AA provided conceptual advice and revised the manuscript. KS designed the review structure, provided conceptual advice, and revised the manuscript. All authors read and approved the submitted version.

## Conflict of Interest Statement

The authors declare that the research was conducted in the absence of any commercial or financial relationships that could be construed as a potential conflict of interest.
