# Corrigendum: Long Non-coding RNA Structure and Function: Is There a Link?

**DOI:** 10.3389/fphys.2019.01127

**Published:** 2019-09-03

**Authors:** Anna Zampetaki, Andreas Albrecht, Kathleen Steinhofel

**Affiliations:** ^1^King's British Heart Foundation Centre, King's College London, London, United Kingdom; ^2^Faculty of Science and Technology, Middlesex University, London, United Kingdom; ^3^Department of Informatics, King's College London, London, United Kingdom

**Keywords:** non-coding RNA, lncRNA, RNA structure, gene editing, cardiovascular diseases

In the original article, there was a mistake in Table 1 as published. A previous version of the Table was published that was not revised and did not include updated references. The corrected Table 1 appears below.

**Table 1:** Structural Determination of lncRNAs.

**LncRNA (size)**	**Mode of action**	**Function**	**Structure**	**Probing techniques**	**References**
Xist (17,000 nucleotides)	*cis*	X-chromosome inactivation.	Regions A-F with distinct repeat sequences.	*In vivo* and *in vitro* SHAPE-MaP. Targeted structure Seq. PARIS	Simon et al., 2013; Fang et al., 2015; Lu et al., 2016; Smola et al., 2016
RepA (1,600 nucleotides)	*cis*	Encoded by an internal promoter on the Xist gene sense strand.	Three folding modules.	*In vitro* using chemical probing with SHAPE and DMS reagents.	Liu et al., 2017a
Rox1 (3,700 nucleotides) Rox2 (1,200 nucleotides)	*cis* and *trans*	Male specific nuclear RNAs. Dosage compensation.	Rox1: three stable helices connected by flexible linker regions. Rox2: two clusters of tandem stem-loops.	*In vitro* using chemical probing with SHAPE and PARS analysis. Both methods independently support the rox2 structure model.	Ilik et al., 2013
SRA (870 nucleotides)	*trans*	Interacts with SRA protein to regulate cardiac muscle differentiation.	Four distinct domains.	*In vitro* SHAPE and DMS chemical probing. Good agreement with RNase V1 enzymatic probing.	Novikova et al., 2012
HOTAIR (2,148 nucleotides)	*trans*	Associated with sporadic thoracic aortic aneurysm and non-end stage heart failure. Circulating biomarker for acute myocardial infarction and congenital heart diseases.	Four structural modules.	*In vitro* using chemical probing with SHAPE and DMS reagents.	Somarowthu et al., 2015; Greco et al., 2016; Gao et al., 2017; Guo et al., 2017; Jiang et al., 2018
Braveheart (590 nucleotides)	*trans*	Cardiovascular lineage commitment.	Three domains. Critical structure: a 5′ asymmetric G-rich internal loop (AGIL).	*In vitro* using chemical probing with SHAPE and DMS reagents.	Xue et al., 2016

The authors apologize for this error and state that this does not change the scientific conclusions of the article in any way. The original article has been updated.
